# The growing pains of physician-administration relationships in an academic medical center and the effects on physician engagement

**DOI:** 10.1371/journal.pone.0212014

**Published:** 2019-02-13

**Authors:** Eric J. Keller, Brad Giafaglione, Howard B. Chrisman, Jeremy D. Collins, Robert L. Vogelzang

**Affiliations:** 1 Center for Bioethics & Medical Humanities, Feinberg School of Medicine, Northwestern University, Chicago, IL, United States of America; 2 HuNamics, LLC, Palo Alto, CA, United States of America; 3 Physician Engagement, Northwestern Medicine, Chicago, IL, United States of America; 4 Department of Radiology, Feinberg School of Medicine, Northwestern University, Chicago, IL, United States of America; University of Indianapolis, UNITED STATES

## Abstract

**Background:**

Physician engagement has become a key metric for healthcare leadership and is associated with better healthcare outcomes. However, engagement tends to be low and difficult to measure and improve. This study sought to efficiently characterize the professional cultural dynamics between physicians and administrators at an academic hospital and how those dynamics affect physician engagement.

**Materials and methods:**

A qualitative mixed methods analysis was completed in 6 weeks, consisting of a preliminary analysis of the hospital system’s history that was used to purposefully recruit 20 physicians across specialties and 20 healthcare administrators across management levels for semi-structured interviews and observation. Participation rates of 77% (20/26) and 83% (20/24) were achieved for physicians and administrators, respectively. Cohorts consisted of equal numbers of men and women with experience ranging from 1 to 35 years within the organization. Field notes and transcripts were systematically analyzed using an iterative inductive-deductive approach. Emergent themes were presented and discussed with approximately 400 physicians and administrators within the organization to assess validity and which results were most meaningful.

**Results & discussion:**

This investigation indicated a professional cultural disconnect was undermining efforts to improve physician engagement. This disconnect was further complicated by a minority (10%) not believing an issue existed and conflicting connotations not readily perceived by participants who often offered similar solutions. Physicians and administrators felt these results accurately reflected their realities and used this information as a common language to plan targeted interventions to improve physician engagement. Limitations of the study included its cross-sectional nature with a modest sample size at a single institution.

**Conclusions:**

A qualitative mixed methods analysis efficiently identified professional cultural barriers within an academic hospital to serve as an institution-specific guide to improving physician engagement.

## Introduction

According to a recent Gallup report, only a third of U.S. workers are engaged in their jobs and physicians are not an exception [1]. Physician engagement is loosely defined as the degree to which physicians feel fulfilled and satisfied by their work, supported within their organization, and motivated to continue their work, recommend their organization, and be involved within their organization [2]. This has become a key metric for healthcare leadership as engaged physicians have been shown to be more productive while making less medical mistakes and are less likely to leave the organization [3, 4].

However, physician engagement has been challenging to measure and improve. Current measures include the National Health Service’s Medical Engagement Scale [5] and proprietary tools from consultants like Press Ganey. These surveys are well-equipped to indicate whether an issue exists but not necessarily why physicians do not feel engaged or how best to support them. Perceptions of professional fulfillment and identity are complex, especially among medical professionals, and not easily appreciated without the use of more sensitive qualitative analyses [6]. Only a few such analyses have been performed, but common issues tend to involve inadequate communication, trust, and support of physician leadership [2, 7, 8].

The focus of the present study involved an 894-bed urban academic hospital that is the central facility within an expanding healthcare network. According to institution leadership, internal Press Ganey surveys of the network’s physicians continued to find low physician engagement despite improvement efforts by the administration (This data was not made fully available to the research team). It was hypothesized that professional cultural differences may be impeding those efforts and by better understanding the cultural dynamics within the organization, one could design more targeted and effective interventions to improve physician engagement. Thus, a qualitative mixed methods approach was devised to efficiently characterize the cultural dynamics between physicians and administrators within the network’s central hospital.

## Materials & methods

This investigation was reviewed by the Northwestern University Social and Behavioral Sciences IRB (STU00206189) and deemed to be exempt from full review. Nevertheless, the risks and benefits were discussed with all participants, stressing the anonymous nature of the analysis, and informed verbal consent was obtained and documented. A novel approach called HuNamic Analysis (HuNamics, LLC, Palo Alto, CA) was used to perform a sensitive characterization of professional cultural dynamics in 6 weeks. HuNamic analysis entails an efficient, customized mixed methods analysis based on the qualitative question(s) of interest. Researchers first work with the research subjects to design a customized approach. Multiple qualitative methods are then used in tandem via an iterative process rather than separately. For example, an interview and observation may be analyzed using grounded theory and phenomenology. Results are compared adjusting and informing each other and prompting further data collection. The authors have found that they arrive at thematic saturation and core themes considerably more efficiently by using multiple methods together in tandem rather than separately.

The core questions in the present study were whether cultural differences between physicians and administrators at the institution were affecting physician engagement, if so, how, and how to improve physician engagement? In initial meetings with leadership, it was clear that this issue had persisted for multiple years in the setting of considerable changes within the healthcare network. Thus, a preliminary analysis was performed of the healthcare network’s history, brand, and written communication to design a more in-depth cross-sectional analysis. The research team searched various permeations of the organization’s name online to assess how the health network was presented publicly and how this had changed over time. Multiple conversations were also held with leadership who shared internal communications and documents that enable a brief look at how the organizational changes had developed and how the environment in which participants worked had changed.

This preliminary analysis suggested that a major shift in the organization had occurred approximately 2 years prior to the study and it would be important to interview and observe physicians and administrators hired prior to as well as after this shift in order to understand the present culture within the organization. This context was also used to design the subsequent data collection and analysis. Semi-structured interviews and observations of physicians and administrators were felt to be sensitive enough to enable a rich understanding of the culture but efficient enough to be useful for the healthcare system. Thus, 20 physicians across specialties and 20 healthcare administrators across management levels were purposely recruited for semi-structured interviews and observation [9]. Participation rates of 77% (20/26) and 83% (20/24) were achieved for physicians and administrators, respectively. Cohorts consisted of equal numbers of men and women with experience ranging from 1 to 35 years within the organization. Participant demographics are provided in Table 1.

**Table 1:** Participant demographics.

Parameter	Administrators (n = 20)	Physicians (n = 20)
Men (%) / Women (%)	10 (50%) / 10 (50%)	10 (50%) / 10 (50%)
Years in the Organization (range)	1–35	1–34
Years of Experience	3–35	1–34
Educational Backgrounds		
M.D. / D.O.	4 (20%)	20 (100%)
M.B.A.	9 (45%)	1 (5%)
R.N.	6 (30%)	0 (0%)
Other Graduate Degree	5 (25%)	8 (40%)
Bachelor (as highest degree)	5 (25%)	0 (0%)

Note: Some participants are recorded more than once under Educational Backgrounds for having multiple degrees with the exception of the row for bachelor’s degree as the highest degree achieved.

The key research questions further informed by the preliminary analysis seemed to require an understanding of social structure, how this structure is internalized/conceptualized by professionals, and how these conceptualizations are expressed, affecting the social structure. Thus, the research team used constructivist grounded theory [10], and phenomenology [11], and discourse analysis [12] in tandem to understand these, respectively. The interview script and observations were designed to facilitate all three of these analytic methods. Interviews began with rapport building discussions about what participants do and how they came to do what they do at the organization. Participants were then asked more sensitive questions about physician-administration relationships and views of the organization using follow up questions such as “why do you think that?” or “what do you think drives that?” to elicit further detail. Participants were also asked about means of improving physician-administration relationships and ideal relationships, roles, and values. The interview script is provided in S1 Table.

Interviews were transcribed verbatim and analyzed using NVivo 11.0 software (QSR International Inc, Burlington, MA). Data collection and analysis occurred simultaneously using an iterative inductive-deductive approach. Constructivist grounded theory, phenomenology, and discourse analysis were performed on a given transcript with field notes of observations, guiding additional data collection. This process is common in grounded theory, but often mixed methods are applied in series with results presented and discussed separately. HuNamic analysis instead applies mixed methods in tandem to arrive at consistent integrated results more efficiently. The disadvantage of this approach is that it prohibits review and discussion of the final results of the separate methodologies. For example, grounded theory coding quickly revealed that communication was a key but diverse concept. Phenomenology and discourse analysis were then applied to better understand how a given participant or group of participants conceptualized and expressed communication. This helped guide axial coding and understanding of communication within the theoretical model developed from the grounded theory analysis. Thematic saturation, the point where additional data stops revealing new themes, occurred after approximately 10 interviews in each cohort, but all 40 participants were interviewed and analyzed given the heterogeneity of their professional roles and experience as well as the use of novel analytical approach. The final coding structure is provided in S2 Table.

All interviews and analyses were performed by a single researcher with 3 years of experience conducting similar studies of professional identity within healthcare. This researcher was neither a physician nor administrator at the organization but did work in healthcare allowing the researcher to establish rapport without bias toward either group. The use of a single researcher ensured a consistent approach to data collection and analysis; however, it exposed the analysis to considerable bias. Therefore, preliminary results were repeatedly presented to and discussed with groups of physicians and administrators within the healthcare network to provide critical feedback regarding the validity of observations and conclusions. In total, results were presented to and discussed with approximately 400 physicians and administrators over a period of 6 months. The results and discussion below are informed by what these physicians and administrators found to be most valid and helpful for improving their understanding of physician engagement.

## Results

This analysis revealed that cultural differences were affecting physician engagement and were potentially the primary driver of low physician engagement scores obtained in previous internal surveys. These cultural differences were primarily affected by organizational changes, conflicting perceptions of these changes related to differences in professional culture, and conflicting meanings behind seemingly shared solutions.

### Organizational growth pains

**Fig 1:**
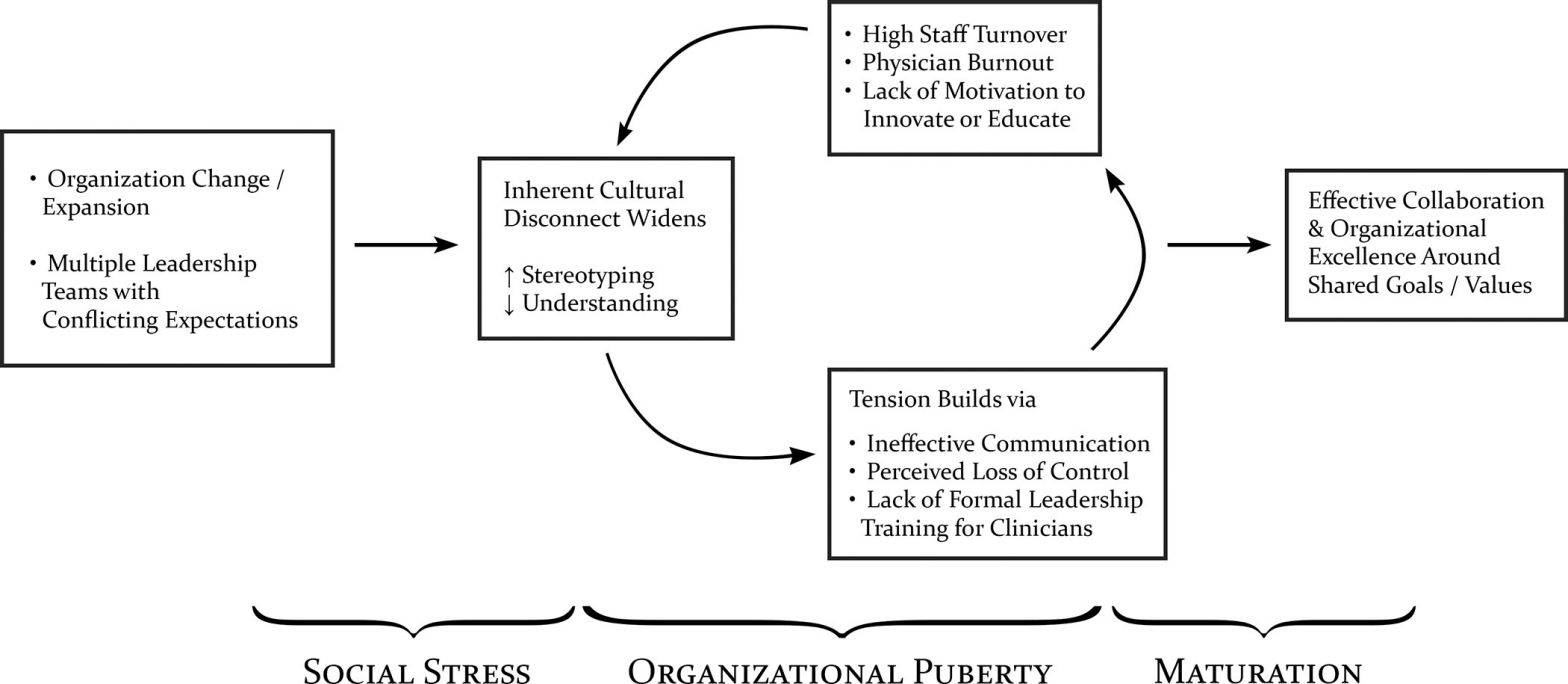
Schematic of organizational growing pains. Changes create social stress, widening cultural differences. Tension can build, deepening the exiting divide. If the organization is able to respond to this, it may mature and rise to achieve shared goals.

The healthcare system had historically been two separate entities, a strong business group (the hospital) and strong physician practice (university faculty physicians), which were merged under a single brand while acquiring other community healthcare facilities. This change exacerbated existing tensions between physicians and administrators and was likened by some to “puberty” or “growing pains.” Fig 1 shows a schematic of this social process. The common perception among physicians was that they had more control and involvement in leadership with the previous model and this was forcefully taken away from them. However, other participants noted that this sense of power was a facade and not different after the merger. These dynamics were further complicated by the facility being an academic center with many physicians having academic interests and duties. Thus, a key frustration among physicians was feeling pulled between academic, clinical, and administrative tasks but having their compensation tied primarily to their clinical productivity. Specialties that generated more revenue and were less hospital-based (e.g. cardiothoracic surgery) felt better supported than specialties such as radiology, anesthesiology, and pathology conveying a sense that the system was more of a business that happens to be healthcare than healthcare that needs business support to run.

“It seems like the only things that ever get done properly are the things that make money for the administration. …I think people have to want it to change. And honestly, I don't think the administration wants or needs it to.”–Physician 03

“If you get really great people, you will build [a great academic healthcare organization] organically. If you treat people like a commodity, you start becoming more kind of like a [large healthcare organization in western U.S.], which is fine and helps people, but it's certainly not going to get you to the number one rankings in academics. It's a different goal.”–Physician 05

### Perceived issues

The majority (90%) of participants described difficulties connecting and collaborating with members of other professional groups, whereas 10% did not perceive a cultural disconnect or disassociated themselves, e.g., concluding it was someone else’s problem. Perceived issues involved recent changes that were well-intended from an organizational care perspective but complicated physicians’ workflows and abilities to engage in academic activities. Examples included a centralized scheduling service, work relative value unit (wRVU) based compensation plan, and reduction of support staff to contain costs and are listed in Table 2 with example quotes from participants. Other common issues discussed included a lack of transparency, ineffective meetings, and lack of effective physician leaders. Physicians tended to note a need for greater involvement in administrative decisions.

**Table 2:** Conflicting perceptions of changes within the healthcare system.

	Positive Perception	Negative Perception
**Compensation Plan**	“…I think the best gift we ever gave to the physicians here is their RVU-based comp plan. Some were upset at first, but the more enlightened ones realized that they no longer had worry about insurance or other matters. They could just focus on doing what they do…”–Administrator 20	“I think [the admins] lost sight of the value of teaching. Now, med students come to my clinics once every two weeks. It's great. I love having them there. But I have to see 8 to 12 patients in my afternoon clinic. You can't say, "Well, I've got this student coming this day, so only schedule 6 to 8 patients or I’ll have to explain to my wife why my pay is being cut next year.”–Physician 14
**Centralized Call Center**	“Now there's a centralized scheduling system. It's one number, one pool of people. If the call center gets 60,000 calls and you had two mistakes this week, if you think about an error percentage. They're human; that's not the worst of errors.”–Administrator 8	“Do they know us? Of course not, how could they know hundreds of physicians? They don't know whether we specialize in this or that, and sometimes people are scheduled completely wrong…. If someone cancels, anybody with a random condition will take that spot even though it could be something completely idiotic for me to see.”—Physician 11
**Support Staff**	“We went through a phase where our cost per case was going up at such an alarming rate, we were going to be in trouble financially… you get that down by reducing resources, and a lot of time those resources are people. So we reduced a lot of human beings and waited to see where the need was before infusing staff back in”–Administrator 7	“Our pulmonary function technician told us she was leaving. She gave us about 3-months’ notice. They didn't even post the position till she was gone which means we're almost a year without another pulmonary function tech… I can't get anybody to understand this is clinically unacceptable, and hire more folks.”–Physician 7
**Engagement Survey**	“I think the engagement stuff, it shows the good intention of the upper administration staff.”–Administrator 19	“One of the biggest things that we struggle with is what our survey results even mean and how to act on them.”–Clinician-administrator 12
	“The good thing about doing the survey is I think we've taken a step back to say how are we really interacting and treating people, our physicians, our employees.”–Administrator 6	“We were told "Go out and fill out the surveys, but don't put what you put last time, you have to realize it's not hurting the people you think it's hurting, it's hurting us.”–Physician 3
**Physician Lounge**	“The physician lounge—wonderful. I mean, I’ve met more surgeons face-to-face in the last two months, I mean more direct relationships than I have the previous five years.”–Physician 17	“"Physician lounge?" I eat lunch at my desk every day. I have no time to go like sit and chat it up at the physician lounge. Who is using the physician lounge? It's certainly not the full-time faculty, we're all completely overwhelmed!”–Physician 7
	“It's lovely. I mean, it's a lovely lounge. I'm glad they did it.”–Administrator 6	“They pay lip service us with these give-away outreach stuff… classic corporate.”–Physician 11
**Maternity Leave**	N/A	“I found out on my maternity leave that I would have to make up any RVU productivity for when I was out, so when you come back, not only do you work full-time, you have to work double that in order to meet your RVU requirement.”–Physician 20

“If you look at [list of top academic medical centers] in almost all of them the lead person is a physician. I think part of it is the leadership's acceptance that a physician has the capability of doing both. Right now, I don't know that [the administration] fully believes that …”–Clinician-Administrator 12.

However, administrators noted that clinicians often falsely assume the acquisition of leadership skills and business sense, leading to the potential for ineffective clinician-managers. Similarly, some clinician administrators noted the lack of resources for clinicians to acquire these skills: “Nobody ever taught me how to manage people. I don't have a medical director handbook….”

The further removed a member of one professional group was from the other, the more he/she stereotyped the actions and intentions of the other group; and when such an individual had one or two members of another group with which they connected, these individuals were perceived as outliers, e.g., “It’s not you [name], it’s those other administrators.” Conversely, individuals that were well-integrated into both groups, such as clinician-administrators, tended to better understand the perspectives of both groups. However, they also risked not being fully-accepted by either: “I think a lot of those guys are yes men and haven’t been clinicians for years….” Younger physicians, those newer to the organization, and clinician-administrators tended to view issues as similar to other institutions or all they knew and responded to engagement efforts more positively. Conversely, senior physicians who had spent most of their career at the institution tended to view the administration as the problem and engagement efforts as misdirected and symbolic of the disconnect. Example quotes are provided in Table 2.

### Observed cultural differences

Professional cultural differences between physicians and administrators were a major contributor to the perceived issues within the organization. Surprisingly, all participants shared common reasons for pursuing their current roles and described those reasons in altruistic terms of service and wanting to help others. However, physicians’ and administrators’ professional backgrounds, values, and ways of thinking differed considerably and are summarized in Table 3. Physicians underwent similarly intense socialization processes based largely on individual success to become autonomous experts. They also adopted distinct specialty cultures [13] that were often most central to their sense of professional identity, i.e., they perceived themselves more as cardiologists or vascular surgeons than [organization] physicians and achieved success through their legacy as researchers, educators, and/or innovators within their specialty. This seemed particularly true for academic physicians, and so recognition of and support for their activities beyond patient care was imperative for their engagement. Conversely, administrators tended to have more diverse professional backgrounds based more on their abilities to achieve goals as part of a team. Professional success often involved vertical ascension to other roles, and so the uniting professional identity was closer tied to being part of the organization rather than a certain role within it. Their goals tended to involve making the organization run well to take care of more people.

**Table 3:** Cultural differences between administrators and physicians.

Parameter	Administrators	Physicians
*Virtues/Values*	Intellectual honesty, Loyalty, Operations, Humble service, Tenderness/steadiness	
*Background*	More diverse	Similar intense socialization process during medical training
*Identity*	Loyalty to and connection via the organization > occupation. Here to support / make things run smoothly	Loyalty to and connection via specialty > profession > organization. The autonomous expert.
*Goals*	Excellent care by advancing the organization and its name/brand.	Excellent care while advancing profession/specialty via education, research, policy, etc.
*Time Horizon*	Weeks to years	Minutes to days
*Problem-solving*	Tall W-shape. Distill information into multiple options.	Short V-shape. Distill information quickly into single best course of action.
*Professional success*	Vertical ascension as part of team.	Individual legacy as clinician, educator, and/or researcher.

**Fig 2:**
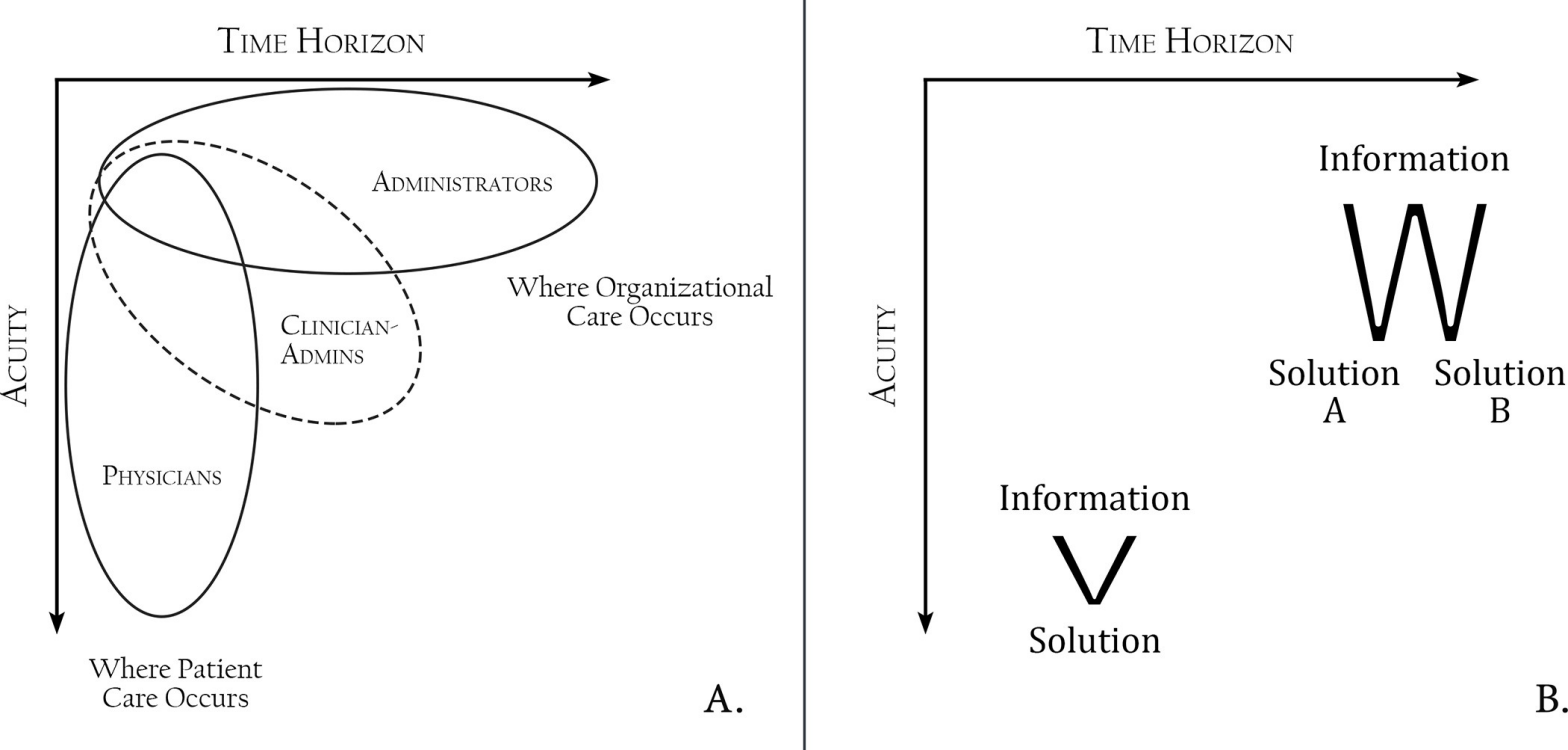
Graphic representation of primary culture divide between physicians, clinician-administrators, and administrators plotted along axes of time horizon and acuity. (A) Shows these divisions in relationship to patient versus organizational care. (B) Shows these divisions as physician-based short V-shaped versus administrator-based longer time horizon W-shaped in relation to decision making.

Another key difference involved groups’ approaches to decision making. Patient care occurred in high-acuity, short-time-horizon environments, and so clinical decision making tended to occur in a short V-shape, where a lot of information was distilled quickly into a single best course of action. Organizational care instead occurred in a tall W-shaped approach, distilling information into multiple possible solutions over a relatively longer amount of time. These differences were reflected in a palpable urgency in interactions with physicians, more chaotic work spaces, and less formal clothing. To administrators, the short V-shaped approach seemed impulsive and less resilient to change: “Physicians don’t like surprises.” To physicians, the W-shaped approach seemed indecisive and excessively bureaucratic. Fig 2 shows schematic representations of these differences.

### Conflicting connotations

Participants who perceived an issue (36/40, 90%) offered similar solutions of needing to increase transparency/communication (36/36, 100%), align incentives (31/36, 86%), and spend more time in each other’s worlds (26/36, 72%); however, a closer analysis of language and context revealed conflicting connotations related to power dynamics and how to implement those solutions. For example, half of administrators and physicians oriented themselves as *bosses* and *islands* where increasing communication meant “getting them onboard” or “making them understand” and presence was about policing others’ activities: “When you leave the kids at home, everybody’s going to act up and do what they want….” (administrator referring to physicians) The other half of administrators and physicians oriented themselves as *partners* and *leaders* where communication and presence were meant to foster understanding: “I think the secret is really caring… trying to be present.” Both physicians and administrators were largely unaware of these differences undermining their efforts to improve engagement.

### Interventions

Physicians and administrators generally responded positively to the presentations and discussions of these results, feeling that they accurately captured their realities and adopting the presented language as a means of discussing issues and challenges. In this way, the presentations of preliminary results became interventions themselves by acknowledging the elephant in the room and providing a forum and language for understanding. Groups of administrators were also presented with strategies to better connect with physicians and establish themselves as partners, e.g., avoiding the word “manage,” approaching conversations with certain specialties different than others, and using physical presence to establish rapport. Bimonthly unstructured meetings were also initiated between different groups of physicians and two senior clinician-administrators that the majority of physicians tended to trust. This allowed physicians to feel “heard” better than previous town hall meetings. Finally, a physician leadership program was initiated to strengthen leadership skills among physicians and position them to take on further leadership roles within the organization.

## Discussion

The results of this study suggest a professional cultural disconnect and perceptions of inadequate leadership were undermining efforts to improve physician engagement. This disconnect was complicated by a minority not believing an issue existed and conflicting connotations despite many participants offering the same solutions for their differences. These issues likely existed previously within the institution but were exacerbated by recent expansion and shifts in management structure that further isolated professional groups and threatened physicians’ sense of professional identity. Below these results are discussed further with how they relate to strategies to improve physician engagement.

Many of these results are well-described by a Social Identity Approach [6] observed previously. Humans have a powerful, innate tendency to form social groups of likeminded individuals, and in doing so, differentiate themselves from others. One tends to view their own group as more nuanced while painting groups in which they are not well-integrated with a broad brush. The more disconnected the groups, the more stereotyping tends to occur. This was observed in the present study where clinician-administrators tended to stereotype less than physicians or administrators relatively isolated from the “other’s” world. Further issues tend to arise if disconnected groups are forced to work together and a group’s professional identity feels threatened. If the threat is not eliminated, it can foster burnout and high turnover or intractable identity conflicts, where one’s sense of identity is based on undermining members of another group, e.g., feeling that being a good physician means standing up to administrators [14, 15]. These processes take time to develop, so it is not surprising that engagement efforts were viewed more skeptically in the present study by physicians who had been within the organizational longer.

Despite these potential detrimental effects of separate professional groups, it is important to realize that it is not necessarily the presence of professional differences that causes issues but how groups respond to those differences [16]. If groups are well-integrated with secure professional identities, these differences in perspectives and experiences can be the lifeblood of innovation and organizational success [7, 17, 18]. Thus, the goal of interventions to improve engagement should not be to eliminate distinct professional groups but to foster understanding and integration. The issue is that fostering role security and group integration is often more challenging than initially perceived. Groups tend to be quick to offer solutions such as increasing communication or teamwork without realizing their conflicting meanings. If present, these seemingly shared solutions can invalidate concerns and reinforce divisions and hierarchies [19]. To facilitate trust and understanding, past authors have suggested training in reflective listening, appreciative inquiry, rounding, values clarification, and making leadership more visible [2, 20, 21]. The hospital in the present study used presentations and unstructured meetings with key stakeholders to facilitate better understanding but could likely benefit from these other approaches.

Many interventions to improve engagement rely on effective leadership to empower others and facilitate a social structure that fosters communication and collaboration [22, 23]. For example, individuals who act as “islands” or “bosses” can be very productive members of a healthcare organization if appropriately placed and managed. However, “leaders” and “partners” are better suited for roles that straddle the worlds of patient and organizational care, e.g., a department chair. By providing resources and programs to develop leadership skills and clear expectations, organizations can engender a community of effective physician leaders and administrative partners that can appropriately support physician leaders or islands. Creating such a community can be particularly challenging for larger organizations where engagement tends to be lower [1] and social dynamics are more complex, but even small improvements in leadership have been shown to significantly improve job satisfaction and reduce burnout among those being led [24, 25]. This is why the hospital in the present study chose to initiate a physician leadership program.

Although the results of this study likely apply to other academic healthcare systems, a similar qualitative study would be necessary to understand how best to improve physician engagement within a given system. Physician-administration relationships have grown increasingly complex over the last few decades as institutions face evolving regulatory pressures and seek to contain costs [2]. It is unclear whether a single management structure is ideal. Instead, a consistent theme seems to be that the most effective structures and approaches come from stakeholders themselves rooted in common values [26–29]. It is difficult if not impossible to understand the cultural complexities of professional groups within an organization with surveys alone, which is why this study used a novel mixed methods approach to efficiently complete a more sensitive qualitative analysis. Such an analysis may be used to design targeted interventions to improve physician engagement as well as organization-specific surveys that better reflect the key issues and language of stakeholders within that organization.

This study had important limitations including its modest sample size of two professional groups relative to the total number of professionals and groups within the organization. However, the sample size was reasonable compared to other studies using grounded theory that tend to achieve thematic saturation within the first 10–12 interviews of relatively homogeneous groups [30]. The single data analyzer was experienced but was also a physician in training, which may have caused a more favorable characterization of physicians. This study was also performed at a single institution making its external validity unclear. Future researchers should consider performing more in-depth comparisons of physician engagement within different medical specialties that are themselves unique cultures. From a macroscopic perspective, it would be helpful to perform a similar multi-institutional comparison with different reimbursement structures and management styles.

In summary, this study used a mixed methods approach to assess the cultural dynamics between physicians and administrators at an 894-bed academic hospital to understand how best to improve physician engagement. The results indicated a cultural disconnect within the expanding healthcare system exacerbated by discrepant meanings and power dynamics not readily appreciated by participants. A series of presentations and discussions with stakeholders was performed to raise awareness and support the need for unstructured meetings with key leaders and a physician leadership program.

## Supporting information

S1 Table Semi-structured interview script. (DOCX)

S2 Table Final coding structure list. (DOCX)
