# Supplementary material for: The growing pains of physician-administration relationships in an academic medical center and the effects on physician engagement
Source: PLoS One. 2019 Feb 13;14(2):e0212014. doi: 10.1371/journal.pone.0212014 (PMC6373942; doi:10.1371/journal.pone.0212014)
Supplement: S1 Table — (DOCX) [file pone.0212014.s001.docx]

**S1 Table: Semi-structured Interview Script**

| Tell me about what you do? How did you come to be in your current role? |
| --- |
| Why did you decide to be a [their position using their words] at [organization]? Have you ever thought about going elsewhere? Why or why not? |
| What’s your relationship like with other [physicians/administrators] here at [organization]? What about with [other group] here? Can you give an example / Why is that / Can you tell me more about that? |
| What do you think the problem is vs. Why do you think that works well? |
| What could make those relationships better? What would an ideal physician-administration relationship look like? Can you give some examples? |
| Does that exist here? What about elsewhere? |
