# Supplementary material for: The growing pains of physician-administration relationships in an academic medical center and the effects on physician engagement
Source: PLoS One. 2019 Feb 13;14(2):e0212014. doi: 10.1371/journal.pone.0212014 (PMC6373942; doi:10.1371/journal.pone.0212014)
Supplement: S2 Table — (DOCX) [file pone.0212014.s002.docx]

**S2 Table: Final Coding Structure List**

| Clinicians as leaders |
| --- |
| Disconnect / Conflicting missions |
| Efficiency vs. Letting things play out |
| Engagement survey as example of disconnect |
| Meetings & committees as wasted time |
| Physician lounge as example of disconnect |
| Generational differences |
| Interests |
| Altruism and humble service |
| Approachable, social skill, awareness |
| Control vs. Lack of control |
| Loyalty to field |
| Loyalty to organization |
| Medical education |
| Operations / Making things run smoothly |
| Recognition beyond financial |
| Research and innovation |
| Role as content expert |
| Role as opportunity to climb corporate ladder |
| Role as physician partner |
| Support staff, resources, demands |
| Transparency / Open communication |
| Perceptions of other institutions (in comparison) |
| Physician identities |
| Manager |
| Fixer |
| Diagnostician |
| Stereotyping / Us vs. them |
| Strategies for improvement |
| Engagement survey as example of support |
| Finding physician champion / advocate |
| Meetings & committees as forum for communication |
| Physician lounge as support |
| Power of presence |
| Being in state of transition / Puberty / Growing pains |
